# Differences in Pulp Wound Healing and Regenerative Potential Between SHEDs and DPSCs

**DOI:** 10.3390/jcm15208162

**Published:** 2026-10-20

**Authors:** Rosa Baldeon-Gutierrez, Masahiko Terajima, Yuria Hori, Ryo Bando, Yuki Kiyokawa, Emi Inada, Yoko Iwase, Tomoya Murakami, Satoshi Watanabe, Naoko Kubota, Hirofumi Noguchi, Masahiro Sato, Issei Saitoh

**Affiliations:** 1 Department of Pediatric Dentistry, Asahi University School of Dentistry, Gifu 501-0296, Japan; ped-yuria@asahi-u.ac.jp (Y.H.); shounishika-bando@asahi-u.ac.jp (R.B.); ykiyokawa@asahi-u.ac.jp (Y.K.); isaitoh@asahi-u.ac.jp (I.S.); 2 Department of Anatomy, Asahi University School of Dentistry, Gifu 501-0296, Japan; terajima@asahi-u.ac.jp; 3 Department of Pediatric Dentistry, Graduate School of Medical and Dental Sciences, Kagoshima University, Kagoshima 890-8544, Japan; inada@dent.kagoshima-u.ac.jp (E.I.); kubotarecta@gmail.com (N.K.); 4 Department of Dentistry for the Disability and Oral Health, Asahi University School of Dentistry, Gifu 501-0296, Japan; iwase@asahi-u.ac.jp; 5 Kyoto Dental Service Center Central Clinic, Kyoto 604-8418, Japan; tomoya.ip@gmail.com; 6 Institute of Livestock and Grassland Science, Tsukuba 305-0901, Japan; watanabe.satoshi225@naro.go.jp; 7 Department of Regenerative Medicine, Graduate School of Medicine, University of the Ryukyus, Okinawa 903-0215, Japan; noguchih@med.u-ryukyu.ac.jp; 8 Department of Genome Medicine, National Center for Child Health and Development, Tokyo 157-8535, Japan; sato-masa@ncchd.go.jp

**Keywords:** dentin–pulp complex, dentin–pulp regeneration, pulp wound healing, stem cells from human exfoliated deciduous teeth (SHEDs), dental pulp stem cells (DPSCs), vital pulp therapies (VPTs), regenerative endodontic procedures (REPs)

## Abstract

Several challenges limit effective pulp wound healing during vital pulp therapies (VPTs) and pulp regeneration in necrotic teeth. Recent advances in regenerative therapies using dental mesenchymal stem cells (MSCs) have shown promise for improving tissue repair and expanding minimally invasive treatment strategies. Among these cells, stem cells from human exfoliated deciduous teeth (SHEDs) and dental pulp stem cells (DPSCs) have gained attention for cell-based VPTs (CB-VPTs) and cell-based regenerative endodontic procedures (CB-REPs). However, little is known about whether SHEDs or DPSCs perform better in CB-VPTs and CB-REPs, particularly regarding wound healing and dentin–pulp regeneration. To date, few studies have compared SHED and DPSC outcomes; separately, CB-VPTs and CB-REPs have shown promising outcomes with both cell types. Dentin bridge formation was generally observed following cell-free approaches, whereas recent in vivo studies of CB-VPTs and CB-REPs have additionally demonstrated the formation of pulp-like tissue. Histological analysis in animal models has identified odontoblasts, angiogenesis, neurogenesis, and connective tissue following CB-REPs using SHEDs or DPSCs. In humans, histological evidence of pulp-like tissue regeneration after SHED CB-REPs has been reported in a single patient. Collectively, preclinical findings and limited clinical evidence suggested that CB-REPs may support injury resolution accompanied by histological regeneration of pulp-like tissue. Both SHEDs and DPSCs represent promising candidates for CB-VPTs and CB-REPs; however, further comparative studies and randomized clinical trials are needed to strengthen the evidence on clinical effectiveness. This review examines the biological properties and regenerative potential of SHEDs and DPSCs within the dentin–pulp complex.

## 1. Introduction

Injuries to the dental pulp—whether from deep caries, trauma, or other causes—can trigger an inflammatory response that, if left untreated, leads to necrosis over time [1]. Achieving pulp wound healing and preserving vitality remain significant challenges in restorative and regenerative dentistry. Although conventional vital pulp therapies (VPTs), such as direct pulp capping (DPC) and pulpotomy, have been recommended by the European Society of Endodontology in cases of pulp exposure and pulpitis in the absence of symptoms [2], they often fail to restore the biological and functional properties of the dentin–pulp complex [3].

Similarly, the use of apexification in immature teeth, despite a high success rate, does not facilitate pulp regeneration or continued root development, leaving the remaining thin dentin walls at high risk of fracture [4,5]. New evidence-based recommendations for immature teeth with apical periodontitis suggest considering revitalization procedures and endodontic tissue engineering [2]. Even for mature teeth, recent publications also support VPTs as a valid, minimally invasive treatment—even in symptomatic mature permanent teeth [6].

**Figure 1:**
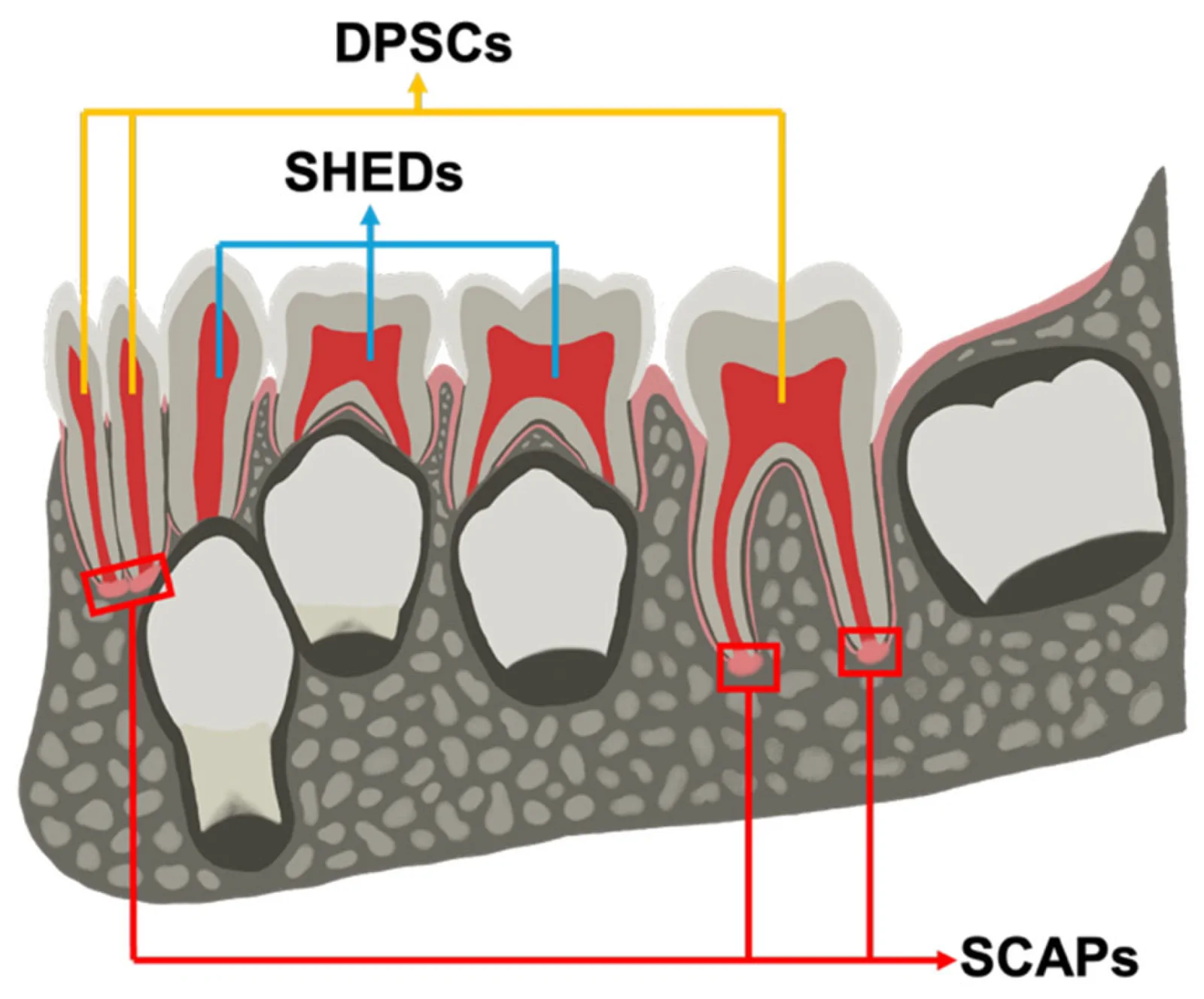
Dental mesenchymal stem cells.

Recent advancements in regenerative dentistry offer promising new alternatives to alleviate symptoms, repair the dentin–pulp complex, and promote neurovascular regeneration, ultimately restoring normal pulp function [7]. Cell-based VPTs (CB-VPTs) aim not only to promote dentin bridge formation at the exposure site but also to support the regeneration of pulp-like tissue within the remaining pulp chamber. In contrast, regenerative endodontic procedures (REPs), which are primarily used to treat necrotic teeth, can be broadly classified into cell-based (CB-REPs) and cell-free (CF-REPs) approaches. CB-REPs involve transplanting exogenous mesenchymal stem cells (MSCs), such as DPSCs or SHEDs, to promote tissue regeneration within the root canal system (Figure 1). Meanwhile, CF-REPs rely on recruiting and/or activating endogenous cells and have historically been referred to as revascularization procedures [4,5,7].

To clarify, ‘repair’ and ‘regeneration’ describe biological outcomes, whereas CF-REPs and CB-REPs describe therapeutic approaches. Cell transplantation refers specifically to the delivery of exogenous cells as part of CB-REPs. Thus, revascularization represents a CF-REP approach based on endogenous cell recruitment. Importantly, the term ‘REPs’ describes the therapeutic approach and does not necessarily indicate complete regeneration of the pulp–dentin complex (Table 1).

MSCs can self-renew and differentiate into various tissue cell types. Within the dental pulp, they are primarily located in the perivascular zone in a quiescent state [8]. Dental MSCs have gained popularity because they can be easily isolated from the dental pulp of discarded teeth, avoiding ethical concerns [9]; however, optimal cell cultivation requires specific conditions, which will be described later. Specifically, stem cells from human exfoliated deciduous teeth (SHEDs) and dental pulp stem cells (DPSCs) are the most commonly utilized in CB-REPs [4,8,10]. In contrast, CF-REPs rely on stem cell migration from the apical papilla (SCAP) into the root via blood clot formation [5] (Figure 1).

SHEDs and DPSCs contribute to pulp wound healing through various biological mechanisms, such as immunomodulation, angiogenesis, and odontoblast-like differentiation [11]. Early research on dental MSCs indicated that SHEDs showed superior proliferative and angiogenic capabilities, along with greater multipotent differentiation capacity. In contrast, DPSCs excelled at generating dentin–pulp-like structures in vivo and exhibited more stable odontogenic differentiation [12,13]. However, recent studies suggest that both cell populations have high proliferative potential, mitigate inflammatory responses after injury, and facilitate regeneration of the dentin–pulp complex [14]. The question of whether DPSCs or SHEDs are the optimal choice for pulp regeneration remains unresolved due to the limited number of standardized clinical studies comparing them.

Consequently, we further compared the regenerative potential and biological properties of DPSCs and SHEDs, with particular emphasis on their roles in pulp wound healing and regenerative endodontic applications.

## 2. Methods

This narrative review summarizes and compares the biological and regenerative properties of SHEDs and DPSCs, with particular emphasis on their potential relevance to pulp tissue repair and regenerative endodontics. We searched PubMed/MEDLINE, Scopus, Web of Science, and Embase for relevant studies published between January 2000 and June 2026.

The search strategy combined terms related to SHEDs and DPSCs with terms describing stemness, proliferation, differentiation, immunomodulation, angiogenesis, odontogenic potential, pulp regeneration, pulp repair, wound healing, and regenerative endodontics. Reference lists of relevant articles and reviews were also manually screened to identify additional potentially relevant studies. Furthermore, we examined key clinical guidelines and the recent literature on VPTs and REPs to place the cellular evidence in its current clinical context.

The PubMed/MEDLINE search included both Medical Subject Headings (MeSH) and free-text terms. Representative search terms included “stem cells from human exfoliated deciduous teeth,” “SHEDs,” “dental pulp stem cells,” “DPSCs,” “dental pulp,” “pulp regeneration,” “pulp repair,” “regenerative endodontics,” “odontogenic differentiation,” “angiogenesis,” “immunomodulation,” and “wound healing.” Equivalent free-text terms and database-specific controlled vocabulary were adapted for the other databases. We performed the final database search in June 2026.

Studies were included if they examined the biological characteristics, regenerative potential, or therapeutic applications of SHEDs, DPSCs, or both, while providing comparative data between the two cell populations. Priority was given to primary experimental studies, direct comparative analyses, studies evaluating DPSC or SHED populations independently, systematic reviews, meta-analyses, and clinically relevant research on pulp preservation or regeneration. In vitro, ex vivo, and in vivo models were considered relevant. Additionally, landmark studies published prior to the specified search period were included if they offered foundational evidence on the isolation, characterization, or regenerative properties of these cells.

Studies were excluded if they were unrelated to SHEDs or DPSCs, focused solely on other dental stem cell populations without relevant data, or lacked sufficient information on the biological or regenerative properties of the cells. Conference abstracts without full text and publications in languages other than English were also excluded.

As this was a narrative review, study selection was based on relevance to the review objectives rather than predefined systematic eligibility criteria. Consequently, the literature was synthesized qualitatively, focusing on consistent and discordant findings between SHEDs and DPSCs. A quantitative meta-analysis was not conducted. While the narrative approach and single-author selection may introduce selection bias and limit reproducibility, these limitations were carefully considered when interpreting the evidence.

To compare evidence across basic, preclinical, and clinical studies, we categorized the literature using a translational evidence framework adapted from established evidence hierarchies. Level 1 comprised in vitro/basic studies; Level 2 encompassed preclinical in vivo animal studies; Level 3 consisted of early clinical evidence, such as case reports and case series; Level 4 involved pilot clinical studies; and Level 5 represented randomized controlled trials. This framework was employed specifically to describe the translational stage of the available evidence, rather than to serve as a formal or validated clinical evidence-grading system.

## 3. Key Biological Differences Between SHEDs and DPSCs

### 3.1. Stemness and Odontogenic Differentiation Potential

Both SHEDs and DPSCs share MSC characteristics, including self-renewal ability and multilineage differentiation potential [15]. However, significant biological differences have been identified between these cell populations (Table 2). SHEDs are considered more immature stem cells because they originate from deciduous teeth, which may explain their higher proliferative activity and greater clonogenic potential compared to DPSCs in vitro [13,16,17]. When stem cell marker expression in SHEDs and DPSCs was compared, SHEDs showed higher expression levels of OCT4, SOX2, NANOG, and REX1 [16] and CXCR4 [9] compared to DPSCs, indicating an increased enrichment of stem cell traits. Similarly, SHEDs showed lower levels of senescence markers than DPSCs [9].

Comparative studies have shown that SHEDs exhibit increased cell proliferation, enhanced mineralization capacity, and stronger angiogenic potential than DPSCs in vitro [17] (Table 2). SHEDs also express elevated levels of growth factors associated with tissue regeneration and vascular formation [17], as well as genes related to cell proliferation and the extracellular matrix—including cytokines such as fibroblast growth factor [18]—suggesting a more active regenerative phenotype.

Studies comparing the odontogenic and osteogenic potential of dental pulp stem cells have yielded conflicting results. Gronthos et al. (2002) reported stable odontoblastic differentiation and robust tissue-specific characteristics in DPSCs [12]. Later, Sabbagh et al. (2020) concluded that while both SHEDs and DPSCs can reconstitute odontogenic tissues, DPSCs showed higher expression of odontogenic markers (DSPP and DMP-1) [10] (Table 2). Conversely, Ziauddin et al. (2022) observed similar mineral deposition in both cell types, as indicated by alizarin red staining [9] (Table 2). More recently, Lu et al. (2025) reported elevated odontogenic differentiation markers (DSPP and DMP-1, ALP and RUNX2) in SHED cultures [19] (Table 2). These discrepancies across studies may reflect differences in anatomical sources.

### 3.2. Angiogenesis and Immunomodulatory Properties

Successful pulp wound healing depends largely on angiogenesis and inflammation regulation within injured pulp tissue to restore vascular supply and ensure cell survival during tissue repair. Inflammation plays a crucial role in triggering pulp stem cells to migrate to the injury site, proliferate, and differentiate into endothelial cells. It also stimulates dental pulp cells and endothelial cells to produce vascular endothelial growth factor (VEGF) and transforming growth factor (TGF-β), thereby aiding dental pulp vessel regeneration [4].

Previous studies have indicated that DPSCs and SHEDs can promote angiogenesis either by direct differentiation into endothelial cells or through paracrine effects [20,21]. Compared with DPSCs, SHEDs showed superior pro-angiogenic activity, as evidenced by increased VEGF secretion [9,20] (Table 2).

Moreover, SHEDs have higher levels of basic fibroblast growth factor (bFGF), which promotes vascular endothelial cell proliferation, and bone morphogenetic protein-2 (BMP-2), a member of the TGFβ family, than DPSCs [22] (Table 2). This enhanced angiogenic capacity may facilitate faster vascularization and improve regenerative outcomes following pulp injury.

Both SHEDs and DPSCs exhibit low immunogenicity and strong immunomodulatory properties, enabling them to suppress inflammatory responses and promote tissue repair [9,23]. They can regulate immune cell proliferation and cytokine secretion and express immunomodulatory and anti-inflammatory factors, thereby contributing to a regenerative microenvironment favorable for pulp healing [11,21].

In vitro studies have shown that when SHEDs are co-cultured with monocytes, they positively influence the differentiation and maturation of monocyte-derived dendritic cells. This process is accompanied by modulation of cytokine secretion, characterized by increased anti-inflammatory cytokines and decreased pro-inflammatory cytokines [24]. Recent in vitro and in vivo studies have demonstrated that when co-cultured with monocytes or M0 macrophages, SHEDs and DPSCs inhibit M1 polarization while promoting M2 macrophage differentiation [25,26,27].

SHEDs have also been reported to reduce pro-inflammatory cytokines and increase antioxidant indices, suggesting potent paracrine effects that reprogram M1 macrophages toward the M2 phenotype [26].

Comparable results in macrophage phenotype modulation were also observed in vivo using exosomes derived from SHEDs [28], opening the door to future studies involving SHEDs or exosomes derived from SHEDs or DPSCs in VPTs. These changes in macrophage polarization are among the immunomodulatory properties of SHEDs and DPSCs and may help resolve infections and injuries, thereby enhancing pulp wound healing and promoting regenerative behaviors.

**Table 2:** Comparison of SHEDs and DPSCs across biological properties and regenerative endodontic applications.

Domain	SHEDs	DPSCs	Comparative Evidence	Key References
Proliferation	Generally high proliferative capacity; several direct comparative studies have reported higher proliferation than DPSCs.	High proliferative capacity, although generally lower than that of SHEDs in several studies.	Direct comparative evidence favors SHEDs in several experimental models.	[17,18,22]
Stemness/stem-cell phenotype	Express conventional MSC/stemness markers; some studies have reported higher expression of pluripotency-associated genes, such as OCT4, SOX2, NANOG, and REX1, compared with DPSCs.	Express conventional MSC markers and retain multilineage differentiation capacity; stemness is well established.	Direct comparative evidence indicates that both have similar characteristics, with some molecular differences favoring SHEDs.	[8,12,13,16,17,18,19,22,29]
Odontogenic differentiation	Strong odontogenic and mineralization potential. Some direct comparative studies have reported greater mineralization or odontogenic differentiation than DPSCs, whereas other studies report comparable or context-dependent outcomes.	Strong odontogenic differentiation potential and established ability to generate mineralized/odontogenic phenotypes.	Direct evidence is available, but the findings are model-dependent rather than uniformly favoring one population.	[10,13,16,17,19,30]
Angiogenesis	Strong pro-angiogenic activity has been demonstrated, including SHED-mediated vascular formation, endothelial differentiation, and exosome-mediated angiogenic signaling.	Strong angiogenic/paracrine activity has also been demonstrated, including enhanced angiogenesis from DPSCs derived from carious teeth.	Evidence is substantial for both populations but predominantly indirect; limited head-to-head evidence prevents a definitive comparison.	[9,17,20,31,32]
Immunomodulation	Extensive evidence demonstrates modulation of dendritic cells and macrophage phenotype, including paracrine and exosomal mechanisms.	DPSC-mediated immunomodulation and macrophage polarization have also been demonstrated, including CCL2-mediated M2 polarization.	Both populations show immunomodulatory activity; however, most studies are independent rather than direct comparisons.	[20,24,25,26,27,28]
Source/donor requirements	Obtained from naturally exfoliating deciduous teeth; collection is generally minimally invasive and does not require extraction of a healthy permanent tooth.	Obtained from permanent teeth, generally requiring extraction or access to pulp tissue.	Clear practical difference; SHEDs offer an accessibility and donor-burden advantage.	[13,16,17,18]
VPT-related evidence	Preclinical evidence supports the ability of SHEDs to promote pulp healing/regeneration, but direct SHED-based clinical VPT evidence remains limited.	Experimental evidence supports DPSC-mediated pulp healing and reparative dentin formation; however, much of the evidence involves animal models rather than direct SHED–DPSC comparisons.	Evidence exists for both, but direct comparative VPT evidence using SHEDs vs. DPSCs is limited.	[33,34,35,36,37,38,39,40]
REP-related evidence	Preclinical studies and case-level evidence have demonstrated pulp regeneration following the transplantation of deciduous tooth-derived cells, including vascularized pulp formation and dentin–pulp regeneration.	A pilot clinical study and subsequent clinical case reports support feasibility of DPSC-based pulp regenerative therapy.	Both have regenerative evidence; however, DPSC evidence is currently more clinically developed in the studies included in this review.	[14,31,37,38,40,41,42,43,44,45]
Histological evidence of regeneration	Histological evidence includes formation of regenerated pulp-like tissue, vascular structures and dentin-like tissues in animal models.	Histological regeneration has been demonstrated in animal models and evaluated in clinical regenerative cell studies.	Preclinical evidence is available for both, but clinical histological evidence remains limited.	[14,31,37,41,42,43,44]
Clinical evidence	Limited clinical evidence in the present literature, including reports of allogeneic SHED use.	More extensive early clinical evidence, including pilot clinical transplantation studies and case reports using autologous DPSC-based therapy.	Current clinical evidence is predominantly early-stage and is not sufficient for a definitive superiority of either cell source.	[37,38,39,40]
Main advantages	Minimally invasive source; naturally exfoliating teeth; high proliferative capacity; strong immunomodulatory and angiogenic potential.	Established dental stem cell population; strong odontogenic potential; extensive preclinical characterization.	The advantages are complementary rather than directly hierarchical.	[13,16,17,18,19,20,22,41,44,45]
Main limitations	Limited clinical evidence; collection requires access to deciduous teeth before exfoliation; biological properties can vary with tooth developmental stage and donor characteristics.	Collection generally requires access to permanent teeth; donor age, tooth condition and inflammation can influence cell properties; clinical protocols remain heterogeneous.	Both populations were affected by donor and protocol heterogeneity.	[16,19,29,30,32,46,47]
Overall translational position	Strong basic and preclinical rationale, particularly for proliferation, angiogenesis, immunomodulation and tissue regeneration; however, clinical evidence remains limited.	Strong basic and preclinical evidence with a comparatively more developed early clinical evidence base for pulp regenerative cell therapy.	Evidence supports complementary translational potential rather than a definitive overall superiority of one population.	

## 4. Evidence in Pulp Wound Healing

Pulp wound healing requires an inflammatory response, immune signaling, and cellular interaction post-injury to achieve regeneration [1]. In healthy individuals, MSCs are recruited to the injury site to differentiate into stromal cells, replacing the damaged ones [1,48]. However, in patients with conditions that impair wound healing, such as diabetes, there is evidence of delayed MSC migration and differentiation [33].

Using DPSCs and SHEDs as a liner in CB-VPTs may enhance or expedite wound healing, particularly in patients where other factors might delay it. As previously mentioned, evidence highlights the significant angiogenic and immunomodulatory properties of these cells; however, their clinical use in CB-VPTs remains limited.

VPTs, such as DPC and pulpotomies, aim to repair damaged areas while preserving pulp vitality. In contrast, the regenerative objectives of CB-VPTs may vary according to the procedure; in DPC, where the space available for new tissue formation is limited, the potential benefit of cell transplantation may primarily involve improving the quality of the newly formed dentin bridge [34,35]. In pulpotomy, the larger space created by removal of the coronal pulp may provide an opportunity to promote the formation of pulp-like tissue, potentially filling spaces not occupied by the dentin bridge [36].

**Figure 2:**
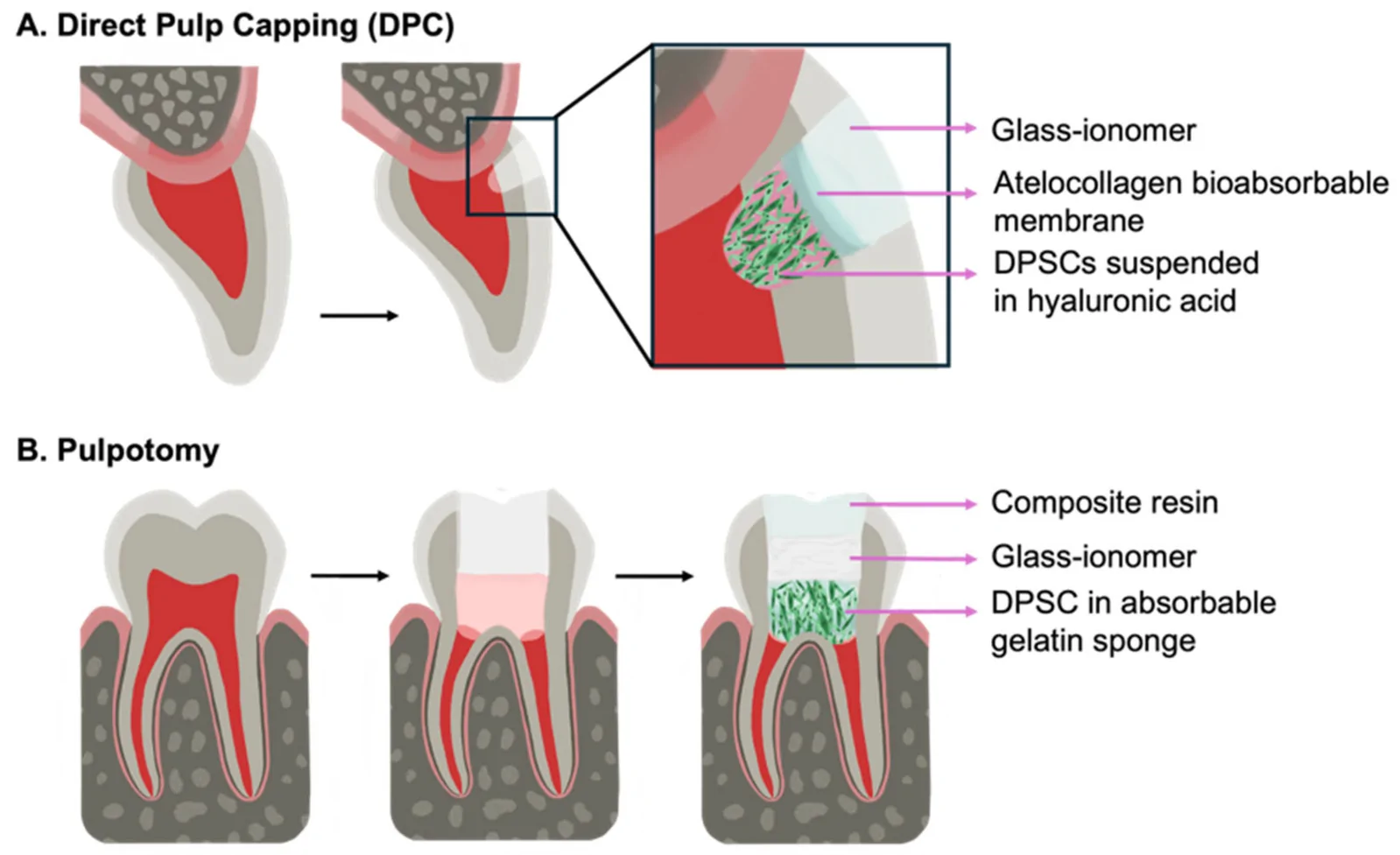
Vital pulp therapies (VPTs).

Two studies (Level 2) on CB-VPTs using autologous DPSCs for DPC in the anterior teeth of dogs reported odontoblast regeneration and complete dentin formation after 3 months, with more favorable outcomes over control groups treated with calcium hydroxide and mineral trioxide aggregate (MTA) [34,35] (Figure 2A). Interestingly, analysis of the newly formed dentin after DPSC capping revealed calcium and phosphorus levels comparable to those of normal dentin, whereas dentin formed in the calcium hydroxide group exhibited lower mineral content [34]. Thus, although both treatments promoted dentin bridge formation, CB-VPTs using DPSCs were associated with more favorable dentin mineralization, suggesting improved tissue quality.

In pulpotomy, the removal of inflamed coronal pulp created additional space in which CB-VPTs may promote the formation of pulp-like tissue beneath the dentin bridge. Evidence from a Level 2 study suggests that DPSC transplantation into the residual pulp after a pulpotomy can enhance tissue formation within this space. In that study, pulp-like tissue occupied 76.8% of the cavity following DPSC transplantation, compared with 47.3% in the control group [36]. Furthermore, the proportion increased to 85.8% when simvastatin, a lipid-lowering medication (hyperlipidemia drug) known to enhance the odontogenic differentiation of DPSCs, was administered [36] (Figure 2B). To date, no studies utilizing SHEDs for CB-VPTs have been reported.

We have previously shown that Level 2 studies demonstrate that macrophages play a crucial role in pulp wound healing; specifically, M2 macrophage polarization is essential for resolving inflammation and proper dentin bridge formation following pulp injury [33]. This, along with several Level 1 studies showing that SHEDs, SHED-derived exosomes, and DPSCs promote M2 macrophage polarization while inhibiting M1 macrophage polarization [25,26,27,28], may partially explain why new pulp-like tissue and better-quality dentin bridges are observed after CB-VPTs. Further studies are needed to better understand the immunomodulatory effects of dental MSCs in pulp wound healing.

Although SHEDs and DPSCs have been investigated in preclinical studies of pulp regeneration, clinical evidence supporting their transplantation for CB-VPTs in which remaining vital pulp is preserved, including DPC and pulpotomy, remains lacking. Higher-evidence-level studies are therefore required to establish the safety and efficacy of CB-VPTs using SHEDs or DPSCs.

## 5. Evidence in Pulp Regeneration

The term revascularization gained prominence in 2001 after a landmark case report by Iwaya et al. (2001) introduced it as a CF-REP for treating immature permanent teeth with apical periodontitis [49]. In 2004, with similar Level 3 reports, Banchs and Trope introduced a modified revascularization protocol involving irrigation and antibiotics [50]. This helped establish the modern clinical technique, which relies on SCAP migration to the root canal after blood clot formation. Since then, numerous case reports have documented favorable clinical outcomes, making it a preferred treatment for immature permanent teeth.

However, Level 2 in vivo studies show that while CF-REPs are effective for tissue repair and soothing symptoms, histological analysis reveals that the hard tissue in the post-treatment pulp resembles cementum-like and bone-like tissues rather than dentin-like tissue, indicating repair rather than true regeneration [42,50].

Conversely, CB-REP Level 1 and 2 studies involving autologous transplantation of SHEDs or DPSCs into immature necrotic permanent teeth have shown regeneration of dentin-like tissue with dentinal tubules in vivo [31,42]. Histological analysis revealed a layer of odontoblasts, blood vessels, and nerves after DPSC transplantation in vivo [31,42].

Level 2 in vivo studies describe odontoblast cells, which are characterized by their typical columnar shape extending into the dentinal tubules [43,44]. Furthermore, a greater reduction in apical diameter was observed in teeth treated with CB-REPs than in those treated with CF-REPs [31,42].

**Figure 3:**
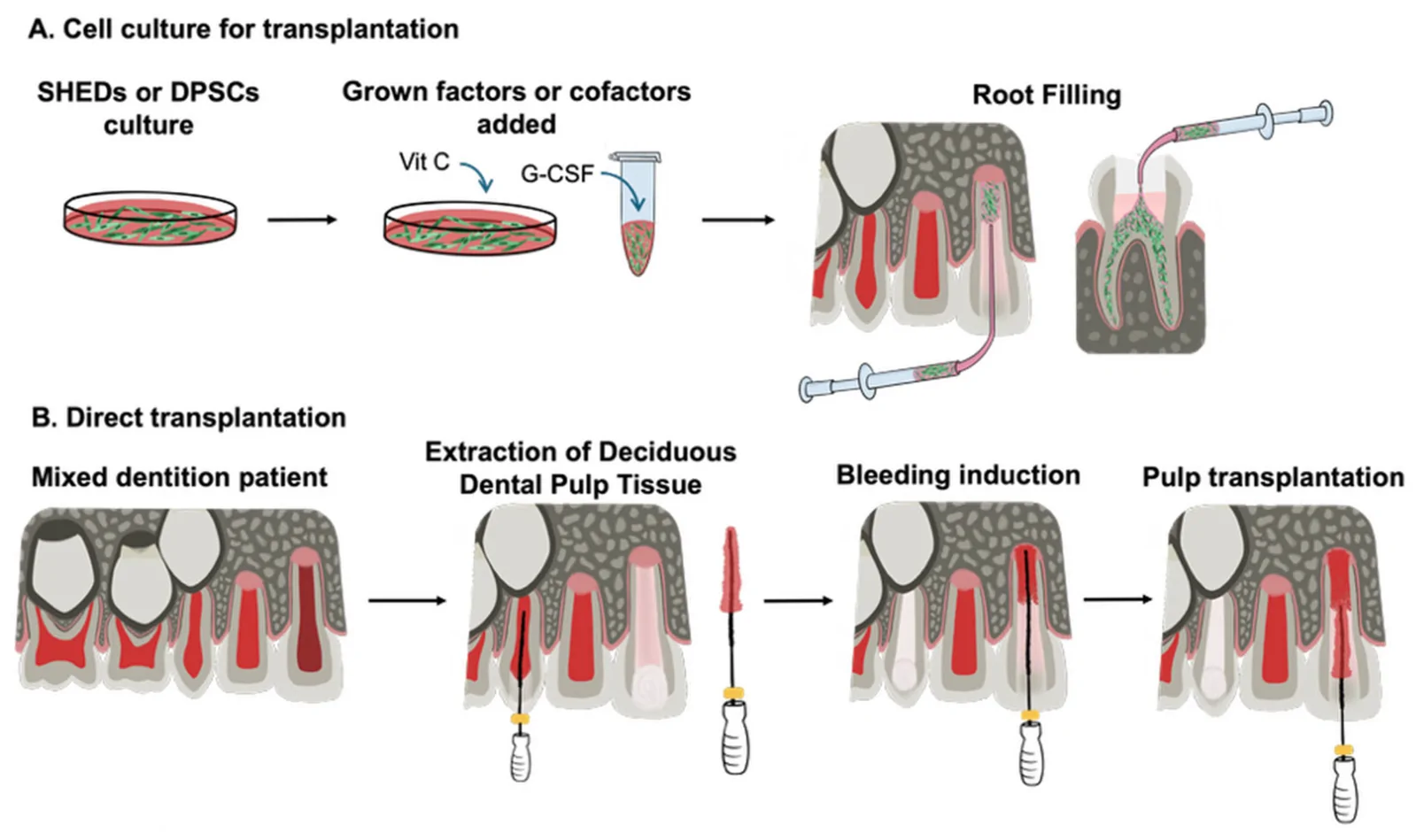
Cell-based regenerative endodontic procedures (CB-REPs).

After confirming promising outcomes in both in vitro and in vivo settings, researchers also conducted pilot clinical studies (Level 4). Among the pioneers was Nakashima et al. (Figure 3A), who performed CB-REPs using DPSCs in patients with irreversible pulpitis in 2017 [37]. Remarkably, three of five patients experienced lateral dentin formation after 24 weeks, evidenced by Cone Beam Computed Tomography (CBCT), while four evaluable patients tested positive on electric pulp testing, indicating restored nerve function in the regenerated pulp [37]. Moreover, magnetic resonance imaging (MRI) follow-up of the five patients showed progressive changes in signal intensity within the root canals after transplantation, with values approaching those of normal pulp at 24 weeks [37]. Although these findings were consistent with the formation and maturation of pulp-like tissue, the absence of histological validation limits confirmation of complete pulp regeneration.

In 2018, a randomized clinical trial (Level 5) by Xuan et al. (2018) (Figure 3A) evaluated CB-REPs with SHEDs in necrotic immature permanent teeth [14]. During 12-month follow-up, CBCT revealed significantly greater root elongation, increased dentin thickness and great apical closure in the SHED transplantation group than in the apexification control group, indicating that the newly formed pulp-like tissue worked normally to support continued root development [14]. Doppler flowmetry and electric pulp testing also demonstrated significant differences between groups, providing evidence consistent with vascularization and sensory responses in the treated teeth. Histological examination of the regenerated pulp tissue of one treated tooth, which was obtained following re-trauma, revealed pulp-like tissue containing an odontoblast layer, connective tissue, blood vessels, and nerves. Although these findings provide direct histological evidence of pulp-like tissue formation in one case, regeneration could not be histologically confirmed in the remaining patients [14].

Although VPTs in mature permanent teeth are not yet widespread, a recent review by Teja et al. (2024) reported the use of a CF-REP for mature teeth called “Seal Bio” [51]. This technique is based on revascularization after enlargement of the apical diameter, followed by intentional over-instrumentation to induce bleeding. Similarly, VPTs (such as DPC and pulpotomy) in mature teeth have been recommended as an alternative approach, even for symptomatic teeth [6].

However, like immature teeth, the newly regenerated tissue after CF-REPs in mature permanent teeth may also lack dentin-like tissue. One Level 2 study explored the potential of CB-REPs for regeneration in dog teeth with closed apices. Using CB-REPs with DPSCs enabled observation of odontoblast-like cells in the newly regenerated pulp-like tissue that were positive for nestin, osteopontin, and osteocalcin. Angiogenesis and neurogenesis were confirmed in CF-REPs and CB-REPs, whereas dentin-like tissue was confirmed only in CB-REPs with DPSCs [45].

Additionally, Level 3 studies using autologous DPSC transplantation in two patients in their 20s with irreversible pulpitis of maxillary molars [38] and in one 44-year-old patient with apical periodontitis of a premolar [39] reported clinical and radiographic success. Treated teeth showed responses similar to those of adjacent healthy teeth on electric pulp testing and MRI [38,39]. This evidence suggests that CB-REPs are not limited to teeth with open apices but may also apply to teeth with fully mature roots, including multi-rooted teeth.

### 5.1. Scaffolds Used for CB-REPs

Scaffolds are three-dimensional biocompatible materials that create a biological environment and provide structural support to facilitate cell proliferation and the development of functional tissues [7,48].

Abdelaz et al. (Level 2) performed DPC using cultured DPSCs without a scaffold, applying them directly to the exposure sites [34]. Another Level 2 study used hyaluronic acid to suspend DPSCs, serving solely as a delivery vehicle [35] (Figure 2A), while in a pulpotomy study (Level 2), Jia et al. co-cultured DPSCs with absorbable gelatin sponge for 1 h at 37 °C before transplantation into the pulp chamber [36] (Figure 2B).

In transplantation, collagen is a popular scaffold material due to its biocompatibility, permeability, and biodegradability, which support cell migration, adhesion, proliferation, and differentiation [48]. A type I atelocollagen sponge was used in Level 2 studies to implant DPSCs [43,44] and to deliver exosomes derived from SHEDs into root canals in vivo [52]. The degradation time of the collagen sponge depends on its size and typically ranges from 8 to 20 weeks [43].

Growth factors have been shown to support signaling processes during the formation and regeneration of the dentin–pulp complex; therefore, they are often used as components of scaffolds [48]. In Nakashima et al.’s clinical studies (Level 3), granulocyte colony-stimulating factor (G-CSF) was added to a suspension of DPSCs in atelocollagen. This growth factor enhances cell survival, migration, and immunomodulation, yielding high regenerative potential [38,39] (Figure 3A).

In Xuan et al.’s randomized clinical study (Level 5), SHEDs were cultured for two passages and maintained for 10 days prior to transplantation (Figure 3A). Vitamin C was added to the medium to promote the formation of dense cellular aggregates enriched in extracellular matrix (ECM), making them thicker and easier to handle [14]. Ascorbic acid, or vitamin C, is a metabolic cofactor that stimulates collagen synthesis and ECM deposition, thereby enhancing cell-to-cell adhesion, stem cell viability, and tissue organization [53]. This strategy may improve post-transplant survival and facilitate the formation of structured pulp-like tissue within the root canal space.

Most studies on CB-REPs isolate dental stem cells, culture them in vitro, and subsequently apply them to injured teeth [14,38,39] (Figure 3A). However, in a Level 2 in vivo study using dog models, Huang et al. (2019) did not culture SHEDs separately prior to the CB-REPs [42]. Instead, the tissue was transferred directly from the deciduous to the permanent teeth after inducing bleeding from the apical tissue, eliminating the need for an exogenous scaffold (Figure 3B). This approach combines CF-REP and CB-REP protocols, using the blood clot from the apical area as a natural scaffold for transplanted SHEDs. Nevertheless, patient selection for this protocol is restricted to children in the mixed dentition stage who have a suitable deciduous tooth available for donation [42].

Although few clinical studies and case reports on DPSC or SHED transplantation have reached a consensus on the optimal scaffold, they all emphasize the importance of using materials, growth factors, or metabolic cofactors to facilitate cell adaptation within the empty pulp canal and promote regeneration.

### 5.2. Histological Comparison

Histological evaluation is considered the gold standard for confirming true pulp regeneration following stem cell transplantation, as clinical and radiographic findings alone cannot accurately determine the nature or organization of the regenerated tissue. Therefore, histological analysis remains essential for distinguishing true pulp regeneration from simple tissue repair.

Although clinical studies (Level 3, 4 and 5) using SHED and DPSC transplantations have shown promising functional outcomes in pulp regeneration, direct histological evaluation in human patients remains extremely limited because of ethical and practical constraints [14,37,38,39]. Currently, histological evidence of regenerated human pulp-like tissue following dental stem cell transplantation is scarce. Consequently, most existing histological evidence comes from animal transplantation models and ectopic tooth slice studies using SHEDs and DPSCs. These preclinical studies consistently show the formation of vascularized pulp-like tissue, odontoblast-like cell layers, and dentin-like structures after independent transplantation of either SHEDs or DPSCs [29,31,37,38,39,42,43,44,45,51,52,53,54,55].

Ziauddin et al. (2022) conducted one of the first direct histological comparisons between SHEDs and DPSCs in an autologous transplantation model in dogs (Level 2) [9]. Although SHEDs show higher proliferative and angiogenic activity in vitro, both cell populations produced histologically comparable pulp-like tissue with similar vascularization, reinnervation, and odontoblast-like cell numbers after in vivo transplantation.

Among the limited human studies available, the work of Xuan et al. (2018) (Level 5) stands out as one of the few direct histological evaluations of regenerated pulp-like tissue after SHED transplantation in humans [14]. This study successfully evaluated the newly regenerated pulp-like tissue 12 months post-transplantation, facilitated by one patient experiencing re-trauma to the treated tooth. The tissue showed components of a normal pulp, including blood vessels, nerves, connective tissue, and odontoblasts.

Despite the existence of histological evidence from animal studies, there is still limited evidence in humans. Therefore, more robust evidence is needed to draw stronger conclusions regarding the optimal histological outcomes of CB-REPs.

## 6. Clinical Considerations

Both DPSCs and SHEDs have demonstrated significant potential in dental pulp regeneration, with several reports suggesting they could be viable options for unconventional treatment cases. Even though the available evidence on CB-REPs remains limited, with studies providing Level 3 to 5 evidence, no adverse effects or toxicity have been reported in the studies to date [14,37,38,39]. Safety assessments in clinical studies have included physical examinations, blood and urine tests, and renal and cardiac function, with no clinically relevant abnormalities reported [14,37,38,39]. Nevertheless, the limited clinical evidence and lack of standardized protocols leave important uncertainties regarding treatment procedures, patient selection and long-term safety. Further research is necessary to develop comprehensive clinical guidelines for identifying patients suitable for DPSC or SHED transplantation, as well as standardized procedures to achieve measurable outcomes over time. Additionally, the extent of root canal preparation, the choice of disinfection solutions, and the use of intracanal medication should be further examined to ensure an optimal environment for cell transplantation.

Although both cell types show similar characteristics during pulp regeneration, clinicians should assess each patient’s condition when considering an autologous cell transplant. Although extracting pulp stem cells is generally minimally invasive and obtaining MSCs from discarded teeth seems easy, obtaining DPSCs requires extractable third molars or premolars, which may exclude patients with tooth agenesis. Moreover, several specific conditions are needed for optimal cell cultivation.

Likewise, the timing of deciduous tooth extraction is vital for obtaining SHEDs, as it heavily influences stem cell quality, with the extent of root resorption being a critical factor. Previous Level 1 studies have identified epithelial cells in dental pulp cultures from exfoliated deciduous teeth. While often considered contaminants, these cells have been associated with bacterial contamination, which can occur when the resorbed apex comes into contact with the periodontium [8].

In vitro research (Level 1) has shown no differences in odontogenic markers between SHEDs extracted from the crown and those from the root [54]. However, the proliferative potential and certain stemness markers were found to be higher in SHEDs from the root [54] and in SHEDs from deciduous teeth with 0 or 1/3 root resorption [29]. In contrast, no such regional differences were observed in the characteristics of DPSCs [55]. This indicates that collecting cells from deciduous teeth with some remaining root is preferable, as it not only minimizes potential contamination but also yields higher-quality cells for regeneration.

Level 1 studies suggested that the presence of caries does not seem to be a contraindication for extracting DPSCs and SHEDs anymore. Permanent teeth with both superficial and deep caries have demonstrated that DPSCs extracted from carious teeth possess characteristics similar to those from healthy teeth in vitro, exhibiting enhanced matrix mineralization and increased expression of angiogenic markers [32,46]. Similarly, in vitro studies on SHEDs from deciduous teeth with active carious lesions and inflamed pulp with irreversible pulpitis have shown that these cells maintain a comparable pattern of proliferation, immunophenotypic characteristics, and differentiation potential to those of cells extracted from healthy deciduous teeth [30,47]. Although these cells have only been evaluated in vitro, they show strong potential for future applications; higher-level evidence is needed to confirm that they have the same regenerative potential as their healthy counterparts.

## 7. Future Approaches and Challenges

Long-term storage of DPSCs and SHEDs needs to be standardized globally. Implementing policies such as a centralized stem cell bank would enable long-term storage of patients’ cells, providing future treatment options for both dental and broader medical applications [55]. Few studies have compared SHEDs and DPSCs after extended storage. The most recent in vitro study providing evidence on the quality of these cells after being frozen for over two years was conducted by Lu et al. [19], who showed that SHEDs exhibited higher levels of stemness genes, whereas DPSCs showed higher levels of senescence genes. Based on these findings, SHEDs appear to have superior long-term storage performance; however, longer evaluation periods are necessary.

The feasibility of using allogeneic cells should be further explored. Currently, evidence is limited; to the best of our knowledge, only one case report (Level 3 evidence) has demonstrated symptom improvement in post-traumatic periapical periodontitis, with resolution of radiolucency and a positive response to electronic pulp testing in two patients after allogeneic SHED transplantation [40]. Although the study reports no signs of long-term rejection, it does not provide references on immune compatibility or safety measures. Therefore, the use of allogeneic DPSCs or SHEDs for transplantation must be explored with caution to prevent immunological rejection.

The potential use of extracellular vesicles, such as exosomes derived from DPSCs and SHEDs, as an allogeneic treatment warrants consideration. Because exosomes are not living cells and are less immunogenic, they may offer a safer alternative to allogeneic agents, reducing the risk of rejection or an exaggerated immune response. Furthermore, the ease of storing and transporting exosomes could make them viable commercial products. Studies have shown that exosomes from SHEDs and DPSCs can enhance angiogenesis in vivo for pulp regeneration [52,56] and support macrophage polarization in vitro [28]. Histological analysis of the new tissue two weeks after exosome seeding in type I collagen membrane implantation showed odontogenic differentiation marker proteins [52]. Further analysis is required to confirm the formation of dentin–pulp-like tissue in root canals when SHED-derived exosomes are used in REP.

## 8. Limitations

Several limitations of the current evidence should be considered when interpreting these findings. First, substantial heterogeneity exists among experimental protocols, including differences in cell isolation, culture, expansion methods, cell passage, scaffold selection, cell dosage, transplantation procedures, and outcome measures. These methodological variations make a direct comparison of regenerative outcomes difficult. Furthermore, findings from animal models may not directly extrapolate to human clinical settings due to differences in dental anatomy, disease conditions, immune responses, and regenerative capacity.

The clinical translation of CB-VPTs and CB-REPs also faces several practical and regulatory challenges. Cell isolation, expansion, characterization, and quality control require standardized procedures and, for clinical applications, compliance with appropriate Good Manufacturing Practice (GMP) requirements. The choice between autologous and allogeneic cell sources adds considerations around donor availability, manufacturing, immunological compatibility, cell banking, and logistics. The costs of cell processing, storage, transportation, and clinical delivery can also limit widespread implementation. Furthermore, regulatory requirements, contamination risks, batch-to-batch variability, and poor reproducibility remain major barriers to clinical translation. Long-term safety concerns, such as abnormal tissue formation or other adverse effects following cell transplantation, warrant further investigation.

Clinical evidence remains limited. Most clinical studies of CB-REPs have involved a small number of patients, with scant randomized controlled evidence and relatively short follow-up periods. Moreover, only a limited number of studies have directly compared SHEDs and DPSCs under comparable experimental conditions. Consequently, differences reported between studies using SHEDs or DPSCs should not be interpreted as evidence of the superiority or equivalence of either cell population.

Finally, human histological evidence remains scarce, limiting our ability to determine whether newly formed tissues represent true regeneration of the pulp–dentin complex or merely tissue repair. These limitations highlight the need for standardized experimental protocols, adequately powered comparative clinical studies, longer follow-up periods, and improved histological characterization of regenerated tissues to clarify the relative regenerative potential and clinical applicability of SHEDs and DPSCs.

## 9. Conclusions

Few studies have directly compared the wound healing and pulp regeneration outcomes of SHEDs and DPSCs under comparable experimental conditions. Consequently, the available evidence does not allow for definitive conclusions regarding the relative advantages of either cell type. Therefore, the potential strengths and limitations of SHEDs and DPSCs must be considered based on the current literature.

Both SHEDs and DPSCs show promising regenerative potential for pulp wound healing and regenerative endodontics. Studies utilizing both cell types have reported favorable outcomes in dentin–pulp tissue healing and regeneration across a range of experimental and clinical settings. However, significant variations in experimental protocols, cell sources, scaffolds, outcome measures, and study models hinder direct comparisons, preventing definitive conclusions about their equivalence or superiority.

To determine the relative regenerative potential and clinical applicability of SHEDs and DPSCs, further standardized comparative studies are needed, particularly well-controlled studies with long-term follow-up in both preclinical and clinical settings. Moreover, before CB-VPTs and CB-REPs can be widely adopted in clinical practice, standardized protocols for cell isolation, characterization, expansion, manipulation, delivery, and transplantation must be established and validated.

## Figures and Tables

**Table 1:** Terminology and conceptual framework for repair, regeneration, and regenerative endodontic procedures.

Term	Definition	Exogenous Cells	Main Concept
Repair	Restoration of tissue continuity or function through formation of replacement tissue that may differ from the native tissue.	Not required	Biological outcome
Regeneration	Restoration of tissue with structural and biological characteristics resembling the native pulp–dentin complex.	Not necessarily	Biological outcome
Cell-free regenerative endodontic procedures (CF-REPs)	Biologically based procedures that do not introduce exogenous cells and rely on endogenous cells for tissue formation.	No	Therapeutic approach
Cell-based regenerative endodontic procedures. (CB-REPs)	Biologically based procedures involving transplantation of exogenous cells into the root canal, usually with scaffolds and/or bioactive factors.	Yes	Therapeutic approach
Revascularization	Historical term for a clinical cell-free approach based on intracanal bleeding and endogenous cell recruitment, with emphasis on restoration of vascularized tissue.	No	Cell-free approach
Cell transplantation	Introduction of exogenous cells into the pulp chamber and/or the root canal as a therapeutic strategy for pulp-like tissue regeneration.	Yes	Cell-based approach

## Data Availability

No new data were created or analyzed in this study. Data sharing is not applicable to this article.

## Abbreviations

The following abbreviations are used in this manuscript:

VPTs	Vital Pulp Therapies
MSCs	Dental Mesenchymal Stem Cells
SHEDs	Stem Cells from Human Exfoliated Deciduous Teeth
DPSCs	Dental Pulp Stem Cells
CB-VPTs	Cell-Based Vital Pulp Therapies
REPs	Regenerative Endodontic Procedures
CB-REPs	Cell-Based Regenerative Endodontic Procedures
CF-REPs	Cell-Free Regenerative Endodontic Procedures
DPC	Direct Pulp Capping
SCAPs	Stem Cells from the Apical Papilla
VEGF	Vascular Endothelial Growth Factor
TGF-β	Transforming Growth Factor-β
bFGF	Basic Fibroblast Growth Factor
BMP-2	Bone Morphogenetic Protein-2
MTA	Mineral Trioxide Aggregate
CBCT	Cone Beam Computed Tomography
MRI	Magnetic Resonance Imaging
G-CSF	Granulocyte Colony-Stimulating Factor
ECM	Extracellular Matrix
GMP	Good Manufacturing Practice
